# Fitness, gray matter volume, and executive function in cognitively normal older adults: cross‐sectional findings from the AGUEDA trial

**DOI:** 10.1002/alz.095249

**Published:** 2025-01-09

**Authors:** Andrea Coca‐Pulido, Patricio Solis‐Urra, Beatriz Fernandez‐Gamez, Marcos Olvera‐Rojas, Dario Bellon, Alessandro Sclafani, Angel Toval, Isabel Martin‐Fuentes, Esmée A Bakker, Javier Fernandez‐Ortega, Manuel Gomez‐Rio, Charles Hillman, Kirk I. Erickson, Francisco B Ortega, Jose Mora‐Gonzalez, Irene Esteban‐Cornejo

**Affiliations:** ^1^ Faculty of Sport Sciences, Sport and Health University Research Institute (iMUDS), University of Granada., Granada, Granada Spain; ^2^ Andrés Bello University, Valparaiso Chile; ^3^ Servicio de Medicina Nuclear, Hospital Universitario Virgen de las Nieves, 18014, Granada, España, GRANADA, Granada Spain; ^4^ Northeastern University, Boston, MA USA; ^5^ AdventHealth Research Institute, Neuroscience, Orlando, FL USA; ^6^ AdventHealth, Orlando, FL USA; ^7^ University of Pittsburgh, Pittsburgh, PA USA; ^8^ Faculty of Sport and Health Sciences University of Jyväskylä., Jyväskylä Finland; ^9^ Instituto de Investigación Biosanitaria (ibs.GRANADA), Granada Spain

## Abstract

**Background:**

Understanding the association of different fitness components with brain structure, and further, how possible fitness‐related brain associations relate to executive function is important for developing public health strategies to improve the health and wellbeing of elderly adults worldwide. Thus, we aimed to investigate the association of cardiorespiratory fitness (CRF) and muscular strength measures with gray matter volume (GMV), and to study whether fitness‐related regions of GMV are associated to executive function (EF) in cognitively normal older adults.

**Methods:**

Ninety‐one cognitively normal older adults (71.69±3.91years; 57.14% females) participated in this study from the AGUEDA trial. CRF was measured by 2‐km walking test and 6‐min walking test. Muscular strength was measured by handgrip strength, biceps curl, squats and isokinetic strength tests. T1‐weigthed images were obtain through a magnetic resonance scan. GMV was determined by voxel morphometric analysis. EF score was created as the composite sum of the z scores of 4 cognitive tests. Linear models were used to determine the association between fitness indicators and GMV adjusting for age, sex, education and body mass index and subsequently, fitness‐related GMV regions were associated with the EF score.

**Results:**

CRF did not show any positive association with GMV. Handgrip strength and knee extension strength were positively associated with GMV in 12 regions (p<0.001, *β* 0.4 to 0.8, k 76 to 2776). Squats strength and 2‐km walking time were negatively associated with GMV in two regions (p<0.001, *β* ‐0.3 to ‐0.4, k 99 to 1102). No associations were found between fitness‐related GMV regions and EF (p>0.05, *β* ‐0.05 to 0.17).

**Conclusion:**

Improving muscular strength throughout resistance exercise may be an effective approach to counteract the age‐related deterioration of physical functioning and brain structure, although the executive function implications should be further explored.

